# Fingerprint of local disorder in long range ordered isometric pyrochlores

**DOI:** 10.1038/s41598-017-12544-8

**Published:** 2017-09-25

**Authors:** Laura Martel, Mohamed Naji, Karin Popa, Jean-François Vigier, Joseph Somers

**Affiliations:** European Commission, DG Joint Research Centre-JRC, Directorate G - Nuclear Safety and Security, Postfach 2340, D-76125 Karlsruhe, Germany

## Abstract

The detailed characterization of local order and disorder in isometric A_2_B_2_O_7_ crystalline pyrochlores is of significant importance in view of their wide range and sensitive technological applications. Nevertheless, much remains to be understood concerning their atomic scale structures. Here we specifically pinpoint local order and disorder in four stoichiometric Ln_2_Zr_2_O_7_ (Ln = La, Nd, Sm and Eu) pyrochlores using a combination of three standard easily available laboratory techniques: XRD, ^17^O solid-state MAS NMR and Raman spectroscopy. The evolution of the oxygen sub-lattice identifies specific features (extra ^17^O NMR signals and Raman bands) which undoubtedly reveal local oxygen order and disorder in these stoichiometric long range ordered crystalline pyrochlores. These results complete the understanding of the atomic scale in these stoichiometric pyrochlores necessitating the need for new microscopic structural models.

## Introduction

Crystalline A_2_B_2_O_7_ pyrochlores have been extensively studied as matrices for immobilization of nuclear waste^1–7^ and have a wide range of chemical applications (for e.g.: Li-ion battery^8,9^, photoluminescence^10,11^, laser materials^12^, solid oxide fuel cells^13,14^…). They possess exotic physical properties^15–18^ exuding a strong interest in their modelling and simulation^19,20^. Part of their diversity lies in their high sensitivity to order and disorder which is often associated with the crystalline transition between ordered pyrochlore and the disordered fluorite structures^21–25^. Disorder can experimentally be due to oxygen non-stoichiometry^26^, cation substitution (e.g. solid-solution)^27^, or more extreme conditions such as irradiation^2,4^ temperature or pressure^28^. The order-to-disorder process in pyrochlores is described in the literature as being governed by the energetics of defect formation determined by the ratio of the cation ionic radii RR = ${\rm{RR}}={r}_{{A}^{3+}}/{r}_{{B}^{4+}}$ 1.46 to 1.80^29^. More recently, by using powerful atomistic computer simulation methods^19,30^, it has been defined that a disorder enthalpy above ~2.6eV was an efficient way to identify an ordered pyrochlore structure. In an ordered pyrochlore (see Supplementary Figure 1), there are two different crystallographic oxygen sites, a (O2, 48f) site coordinated by two A and B cations and a (O1, 8b) site surrounded by four A cations. One must also consider the ordered oxygen vacancies (V_O_, 8a) surrounded by four B cations, which are located in the second coordination sphere of the (O2, 48f) sites. In the defective fluorite structure, there is a complete mixing of the cation sites and the 8a site becomes fully occupied by (O3, 8a) oxygen atoms. This leads to a crystallographic structure where there is no more distinction between the (O1, 8b), (O2, 48f) and (O3, 8a) sites and with an occupancy of 56/64 = 7/8 for each site. In between the ordered pyrochlore and the disordered fluorite, some disordered pyrochlores have been described^31,32^.

Our attention has been triggered by the lanthanide zirconate series, and more specifically La_2_Zr_2_O_7_ (RR = 1.61), Nd_2_Zr_2_O_7_ (RR = 1.54), Sm_2_Zr_2_O_7_ (RR = 1.5) and Eu_2_Zr_2_O_7_ (RR = 1.48) which on the basis of defect formation energies^29^ and disorder enthalpies (i.e. above ~2.6 eV)^19,30^ should all be fully ordered pyrochlores. It is worth mentioning that compared to other pyrochlore families, this series nevertheless possess a higher tendency to disordering^19,33^. Interestingly, a literature screening for formally the same stoichiometric Ln_2_Zr_2_O_7_, shows numerous values of the lattice parameters (See Supplementary Figure 2), which is often taken as the main parameter to characterize these materials. Indeed, some parameters as the stoichiometry^26,34^ or synthesis conditions can influence its value, but, this observation clearly questioned the current experimental characterization of their local structures. A technique such as neutron total scattering has recently been very efficient to investigate the local order allowing the identification of domains with the Weberite structure at the local scale within a defected fluorite structure at the large scale^27^. Unfortunately, due to their high neutron absorption cross sections, this technique cannot be applied to the Ln_2_Zr_2_O_7_ with Ln = Sm, Eu and Gd^35^. Though other techniques have been used to probe the local order and disorder in this Ln_2_Zr_2_O_7_ pyrochlore series at different length scales, they have suffered by virtue of their indirect approach and disorder was unveiled by detecting an anomaly in the trend of the resulting parameters over the full series of pyrochlores. Thus, Zr-XANES yields a variation of the Zr-coordination number^35^ and Raman spectroscopy a broadening of the bands^31^. In the present communication, the structural complexity has been unravelled by XRD, to probe the long range order, and ^17^O MAS NMR and Raman spectroscopy to elucidate changes in the oxygen local environment of these four stoichiometric rare earth pyrochlores. Thereby, it was possible to detect directly this important subtle order-to-disorder variation by probing the local O-atom environment. The observations reported here are of acute importance as they form the basis for the complete understanding of what is essentially “an ordered pyrochlore structure”.

## Results

**Figure 1:**
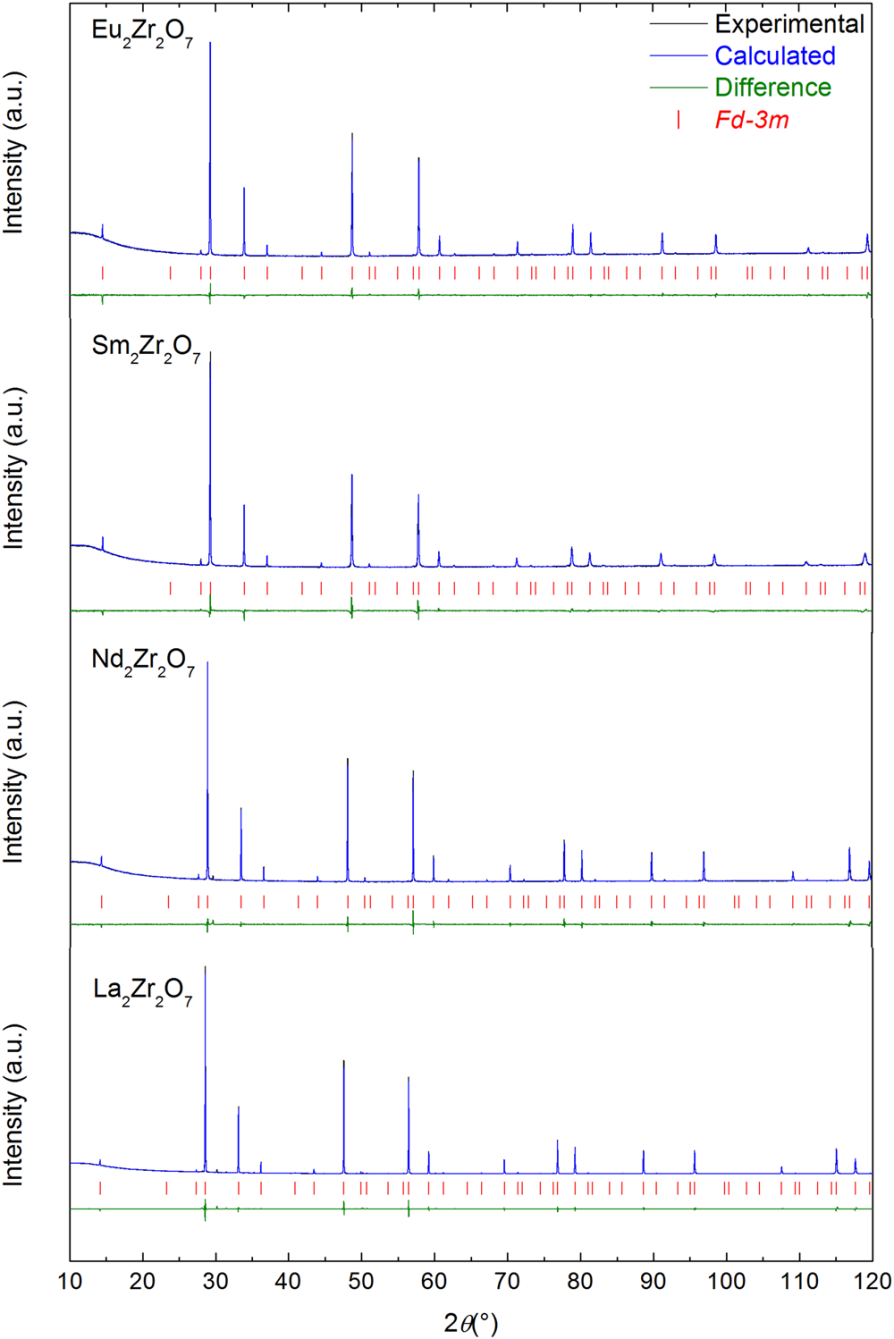
XRD patterns of the Ln_2_Zr_2_O_7_ pyrochlore and their corresponding Rietvield refinement depicting the high crystallinity of the Ln_2_Zr_2_O_7_ samples.

**Figure 2:**
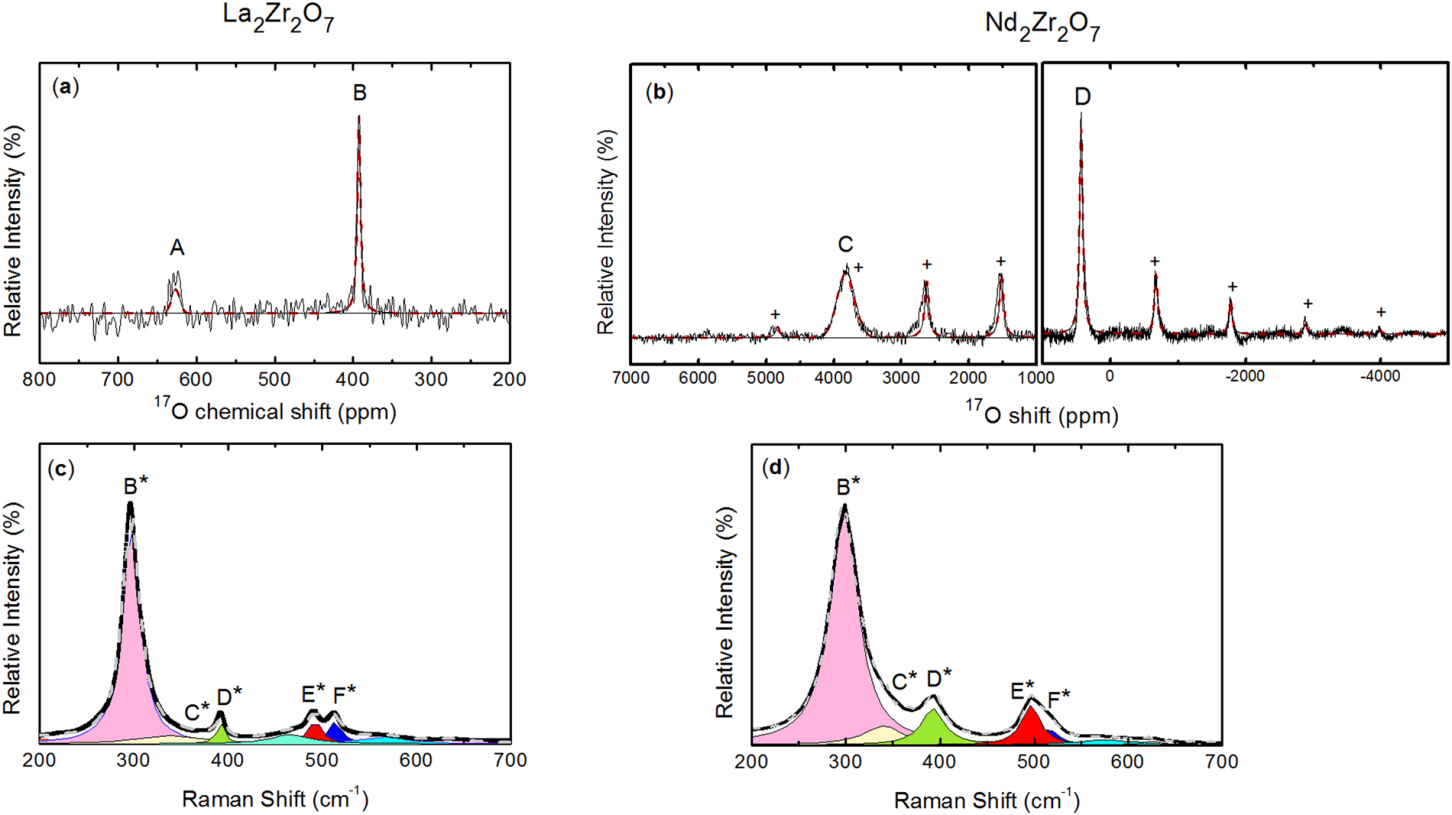
^17^O MAS NMR spectra (black) and their corresponding fits (dashed red) acquired at 60 kHz for (**a**) La_2_Zr_2_O_7_ and (**b**) Nd_2_Zr_2_O_7_ (the crosses show the spinning sidebands). And, the Raman spectra (black) of (**c**) La_2_Zr_2_O_7_ and (**d**) Nd_2_Zr_2_O_7_ and their corresponding fits.

The XRD patterns of the four well crystallized pyrochlores are presented in Fig. 1 confirming their purity (a small non-characterized impurity was identified in the pattern of La_2_Zr_2_O_7_). The patterns possess the characteristic superstructure peaks of cubic pyrochlore, Fd-3m and the lattice parameters of 10.8019(1), 10.6911(1), 10.5753(1) and 10.5533(1) Å were obtained for respectively La_2_Zr_2_O_7_, Nd_2_Zr_2_O_7_, Sm_2_Zr_2_O_7_ and Eu_2_Zr_2_O_7_. These values are in general agreement with the one previously published^26,34,36–44^ and, especially with stoichiometric pyrochlores. As expected, there is an increase of the lattice parameter as a function of the ionic radii (Supplementary Figure 2). As both ^17^O MAS NMR and Raman spectra present similarities for La_2_Zr_2_O_7_ and Nd_2_Zr_2_O_7_, we will describe them together. Their ^17^O MAS NMR spectra depicted in Fig. 2 both possess two ^17^O signals at 623.3 (A) and 388.7 (B) ppm for La_2_Zr_2_O_7_ and at 3890 (C) and 434 (D) ppm for Nd_2_Zr_2_O_7_. In addition, an important set of spinning sidebands due to first order quadrupolar interaction (see Supplementary Figure 3) for peak B (C_Q_ = 121 kHz, η_Q_ = 0.7) and D (C_Q_ = 635 kHz, η_Q_ = 0.8) are detected. This characteristic pattern of spinning sidebands is linked to local distortions around the O site as described elsewhere^45,46^. Therefore Peaks B and D can be both attributed to the (O2, 48f) site and consequently, peaks A and C to the (O1, 8b) site. For La_2_Zr_2_O_7_, this attribution is confirmed by the relative intensity between peaks B and A which is 6:1 as expected from its crystal structure. Even if the ^17^O MAS NMR spectrum of Nd_2_Zr_2_O_7_ is only qualitative (as it was acquired using two offsets), the large ^17^O NMR shift of peak C conforms with O atoms surrounded by four Nd^3+^ spin bearing centres with unpaired electrons (see Supplementary Note 1)^47–50^. The Raman spectra of La_2_Zr_2_O_7_ and Nd_2_Zr_2_O_7_ can both be reproduced by a set of five Lorentzian bands corresponding to the most obvious vibrational modes^28^. They are labelled B*, C*, D*, E* and F* with Raman frequencies at 297, 339, 392, 492 and 513 cm^−1^ for La_2_Zr_2_O_7_ and 298, 339, 392, 497, 515 cm^−1^ for Nd_2_Zr_2_O_7_, respectively. Two extra weak Raman bands at about 464 cm^−1^ and 561 cm^−1^ were necessary to reproduce the shape of the E* and F* doublet in La_2_Zr_2_O_7_ and are very likely due to the impurities detected by both XRD and ^17^O MAS NMR as they were not detected in reference^28^.

**Figure 3:**
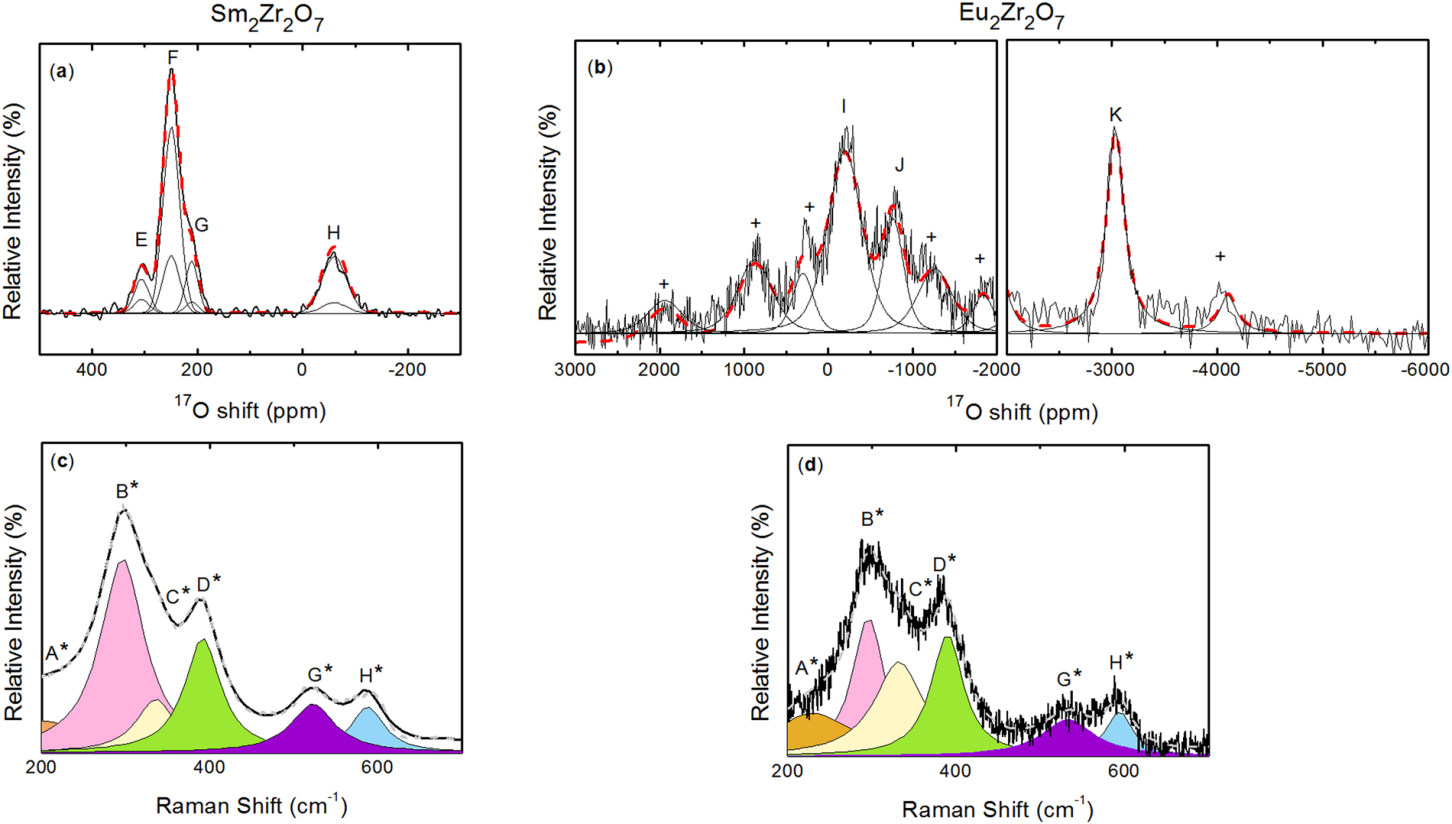
^17^O MAS NMR spectra (black) and their corresponding fits (dashed red) of (**a**) Sm_2_Zr_2_O_7_ and (**b**) Eu_2_Zr_2_O_7_ acquired at 60 kHz (the crosses show the spinning sidebands). And, the Raman spectra (black) of (**c**) Sm_2_Zr_2_O_7_ and (**d**) Eu_2_Zr_2_O_7_ with their corresponding fits.

Interestingly, both ^17^O MAS NMR and Raman spectra of Sm_2_Zr_2_O_7_ and Eu_2_Zr_2_O_7_ presented in Fig. 3 differ strongly from the La and Nd pyrochlores. The ^17^O MAS NMR spectrum of Sm_2_Zr_2_O_7_ possesses four peaks at 306.2 (E), 249.3 (F), 210.8 (G) and −60.3 (H) ppm with relative intensities of 13, 61, 12 and 14%. Due to its ^17^O NMR shift, peak H can be attributed to oxygen atoms in the (O1, 8b) site (See Supplementary note 1) while peak F can only be attributed to (O2, 48f) due to its high relative intensity and important spinning sideband pattern. Taking the most obvious features in the Eu_2_Zr_2_O_7_ spectrum, three main peaks can be identified at −196 (I), −767.5 (J) and −3033.3 (K) ppm. This sample exhibits important spectral broadening due in part to the paramagnetic effect as observed in other Eu-based materials^51–53^. Considering its ^17^O NMR shift, peak K can be attributed to O atoms at the (O1, 8b) site (See Supplementary note 1). Even if the current spectrum is qualitative, peaks I and J are derived from local disorder with one of them emanating from the (O2, 48 f) sites. For both samples, spinning sidebands are detected for all the peaks: peak E (C_Q_ = 1412 kHz, η_Q_ = 0.7), peak F (C_Q_ = 756 kHz, η_Q_ = 0.5), peak G (C_Q_ = 1067 kHz, η_Q_ = 0.6), peak H(C_Q_ = 1067 kHz, η_Q_ = 0.6), peak I (C_Q_ = 874 kHz, η_Q_ = 1), peak J (C_Q_ = 1237.57 kHz, η_Q_ = 1) and peak K (C_Q_ = 528 kHz, η_Q_ = 0.8).

**Figure 4:**
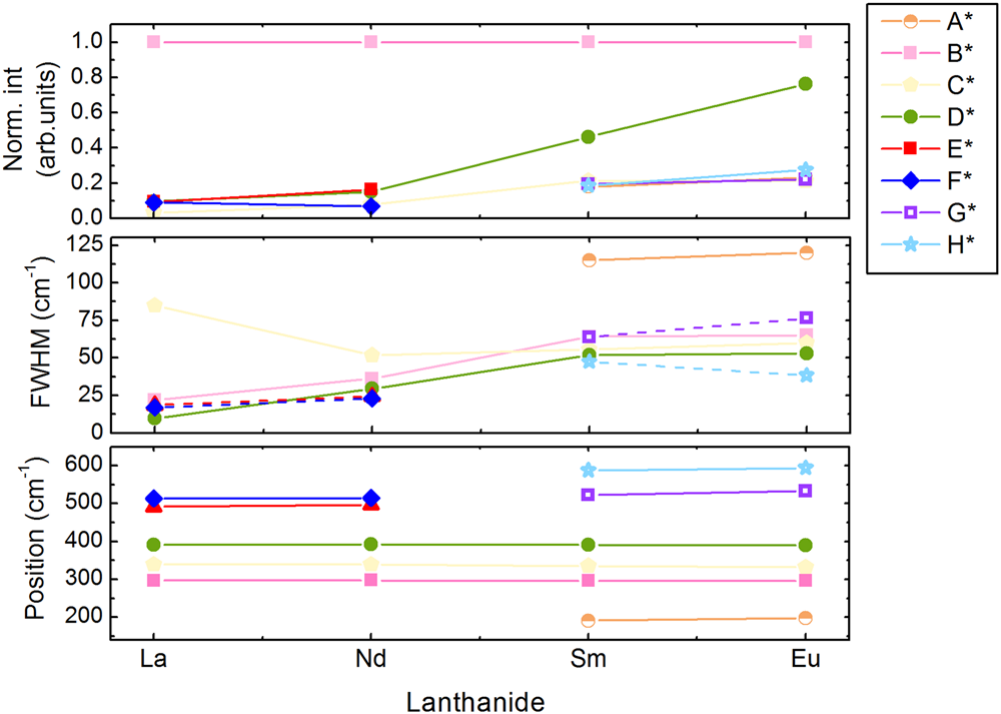
Variation of Raman parameters – band position, FWHM and normalized intensity- as a function of the lanthanide cation. Solid lines serve as guides for the eye. The slightly higher FWHM for band C* in La_2_Zr_2_O_7_ might be due to to the overlapping between this band and the one of an impurity.

The Raman spectrum of Sm_2_Zr_2_O_7_ seems very similar to that of the La and Nd counterparts, but, an additional band must be introduced at 191 cm^−1^ (A*) to reproduce the low frequency tail of the spectrum. The remaining five bands at 296, 336, 392, 523 and 588 cm^−1^ are designated as B*, C*, D*, G* and H*. For Eu_2_Zr_2_O_7_, six bands at 198, 296, 332, 390, 533 and 594 cm^−1^ attributed to A*, B*, C*, D*, G* and H* were also identified, albeit with a substantial increase of the line broadening. The fitted Raman band parameters have been plotted in Fig. 4.

## Discussion

The main finding of this research is the specific identification of local disorder in each of these pyrochlores, whose derivation presented in Supplementary note 2 has been achieved on collective examination of the entire series, as commonly done. We can now start our discussion with ^17^O MAS NMR as it is a proven powerful tool to probe local order and disorder in crystalline pyrochlore^54^. The fully ordered stoichiometric pyrochlore structure possesses only two distinct crystallographic O sites; the presence of more than two O signals on the ^17^O MAS NMR spectrum immediately signifies a local disorder at the atomic scale. This is clearly observed for Sm_2_Zr_2_O_7_ and Eu_2_Zr_2_O_7_ for which no impurities were detected by XRD. As the ^17^O MAS NMR spectrum of Sm_2_Zr_2_O_7_ was acquired under quantitative conditions we give it more attention. The intensity ratio of peaks (E, F, and G): peak H is 6:1, i.e. exactly the ratio expected between the two different crystallographic sites (O1, 8b) and (O2, 48f). The intensities of peak E, G and H are very similar, but cannot be simply ascribed to a depopulation of the (O2, 48f) sites into the vacant (V_O_, 8a) sites, as this would imply full occupancy of the vacancies. Therefore, the mechanism underlying the disordering seems to be more complex than expected for a simple depopulation of the (O2, 48f) sites. This NMR observation could have been due to the presence of a cationic disorder in our samples, but, the fitting of the XRD patterns considering this type of disorder did not improve the Rietveld refinement (not shown). The increase of disorder is also seen when comparing the ^17^O MAS NMR spectra of the two ordered pyrochlores with those of the two disordered pyrochlores, one can notice the apparition of spinning sidebands for peak H and K (contrary to peaks A and C) which correspond to the oxygen at the (O1, 8b) sites. This reveals an increase of the local distortion around the O atoms (See Supplementary note 3)^55^. As the cation size decreases through the series, a steric effect cannot be at the origin of these bond distortions, but in contrast, it can be linked to an increasing disorder in the second coordination shell. A first glance of answer can nevertheless be given to understand this anionic disorder based on the previous Zr-XANES results which showed a change of coordination number through the Ln_2_Zr_2_O_7_ series associated to a growing disorder through the series^35^.

Accordingly, the second and most convincing proof is found in the Raman vibrational mode assignment, a key pointer in the understanding of the competition between order and disorder. Alarmingly, many discrepancies exist in published assignments thereof ^56–58^. The most common method to interpret the Raman data relies on the spectral comparison with each other and between different pyrochlore families^42–44^. Here, we take an alternative approach and attribute the different modes by comparing the spectra through this Ln_2_Zr_2_O_7_ series. The five characteristic Raman bands detected for the ordered La_2_Zr_2_O_7_ and Nd_2_Zr_2_O_7_ have similar frequencies as those previously published supporting the presence of local order and their good stoichiometry^28,59^. Importantly, the energy of all modes seems to be independent of the lanthanide cation except for the E* mode for which a clear upshift is observed when substituting La with Nd (see Fig. 4 and Supplementary Figure 4). This is in good agreement with the atomic displacement analysis^58^ for these modes and supports their attribution to pure oxygen motions, except for the E* mode where a small contribution from Ln^3+^ might be present. Armed with a characterization of the Raman spectrum of these two ordered pyrochlores, we analysed those of the disordered pyrochlores. We want to stress that as the main topic of this paper is to show the presence of local disorder at the atomic scale, we did not attempt a full characterization of the Raman modes. We therefore focus our discussion on modes A*, E*, F*, G* and H* which differ between ordered and disordered pyrochlore spectra. As mode A* appears in the spectrum of Sm_2_Zr_2_O_7_ and Eu_2_Zr_2_O_7_ (its absence is also confirmed in their equivalent ^16^O Raman spectra reported in literature^28,43,44^) it is unlikely that neither involves (O1, 8b) nor (O2, 48f) oxygens, since both sites are fully occupied in the ideal pyrochlore. To better understand the mode A*, we have to pursue our discussion of the other Raman bands. Our main observation is that G* and H* modes correspond to E* and F* modes, but shifted to higher Raman frequencies. Though, the shift from E* to G* can be explained by the decrease of the lanthanide ionic radii (or increase of the force-constant) the 70 cm^−1^ upshift of F* to H* is unlikely to be caused by the same effect. When considering the Raman data for La_2_Zr_2_O_7_ to Nd_2_Zr_2_O_7_, there is the previously discussed upshift of mode E* to slightly higher frequency which continues for Sm_2_Zr_2_O_7_ (Supplementary Figure 4). But, in the case of mode F*, there is no variation of the Raman band energy from La_2_Zr_2_O_7_ to Nd_2_Zr_2_O_7_ which should, according to the same logic, lead to an upshift of mode F* of at least 10 cm^−1^. To support this statement, we took account of the previously published force constant change which confirms that the predicted splitting should not exceed 10 to 20 cm^−1^ ^60^. The difference in frequencies observed here between F* and H* is therefore far too high. Finally, ab initio calculations also seem to predict a mode at 617 cm^−1^ but, unfortunately, for the optically inactive F_1g_ symmetry instead of the F_2g_ symmetry expected for mode F*^61^. Based on all these evidences, we can safely propose that the two well resolved modes E* and F* in Nd_2_Zr_2_O_7_ and La_2_Zr_2_O_7_ actually merge to form the broad band G* for Sm_2_Zr_2_O_7_ and this “merging” is caused by the apparition of the anionic disorder. While mode G* belongs to the ideal pyrochlore structure, it is definitely not the case for mode H* whose observation might be a result of symmetry breaking due to an inherent disorder in the O sites. Therefore, this band is derived from the Zr-O stretching motion modified due to interstitials in the Zr-environment. With this in mind, we can come back on the low frequency mode A* which can be attributed to the vibration of oxygen atoms at the centre of Zr_4_O tetrahedral due to interstitial O atoms.

The important redefinition of both these additional vibrational Raman bands inaccurately assigned in the literature, in addition to the ^17^O MAS NMR analysis, specifically describe the inherent O local order in stoichiometric La_2_Zr_2_O_7_ and Nd_2_Zr_2_O_7_ and disorder in stoichiometric Sm_2_Zr_2_O_7_ and Eu_2_Zr_2_O_7_. It is worth mentioning that considering the previously calculated disorder enthalpy formation (both cationic and anionic)^19,30^, both Sm_2_Zr_2_O_7_ and Eu_2_Zr_2_O_7_ belong to the same range of defect energy formation. Even more interesting for this same family, the Raman spectrum^31^ of Gd_2_Zr_2_O_7_, which also belongs to this range of energy, possesses the specific bands that we presently clarified through the text and characteristic of disordered pyrochlore. These observations might underline the range of energy delimiting ordered from disordered stoichiometric pyrochlore. Finally, our present study can easily be extended to any pyrochlore system but also, implemented to solid-solution or to their modelisation using computational methods. We can safely state that it will help assisting in the construction of a complete understanding of these complex and of high technological importance systems.

## Methods

### Synthesis

Polycrystalline samples of La_2_Zr_2_O_7,_ Nd_2_Zr_2_O_7_, Sm_2_Zr_2_O_7_, and Eu_2_Zr_2_O_7_ were produced by liquid route. For this purpose, exceptionally accurate devices for weight (analytical balances of 5 decimals) and volume (fix volume pipets) measurements have been used. Thus, the possible deviations from the stoichiometry were controlled during all the production flux. Solutions of *Ln* ^3+^ (La^3+^, Nd^3+^, Sm^3+^, Eu^3+^) have been prepared by dissolution in nitric acid *of La*_2_ *O*_3_ *(Alfa Aesar, REO (99.99%), Nd*_2_O_3_ (Alfa Aesar, REO), *Sm*_2_O_3_ (Alfa Aesar, 99.9% metal basis) and *Eu*_2_O_3_ (Merck, purity >99% metal basis), respectively. Zirconyl-oxynitrate solution has been prepared by dissolution of ZrO(NO_3_)_2_·*n*H_2_O (Fluka, 99.99% trace metal basis) in distilled water and its concentration has been quantitatively determined by gravimetry. Appropriate volumes of these solutions were mixed and slowly dried. The residue was calcined in alumina crucibles under air at 900 °C (12 h), 1200 °C (12 h), and 1400 °C (72 h) with intermediate grindings. These temperatures are under the pyrochlore to fluorite order-disorder temperature described in the literature^33,62^. These pure lanthanide zirconates with pyrochlore structure were further enriched in ^17^O as it is the only active nucleus for NMR and possesses a low natural abundance (0.04%)^63^. To do so, the materials were enriched by the gas exchanged (O_2_ enriched at 70% mixed with Ar) technique by putting the powders during 24 h at 800 °C in a furnace. These samples were used for analysis by XRD, Raman and ^17^O MAS NMR in order to avoid the uncertainties led by analysing different samples.

### XRD

The patterns of the three ^17^O-enriched well crystallized samples (Fig. 1.) were obtained at room temperature on 20 mg of a powdered sample. A Bruker D8 Advance diffractometer (Cu Kα radiation, 40 kV, and 40 mA) with a Bragg−Brentano θ/2θ configuration were used for the analysis. This diffractometer is equipped with a curved Ge monochromator (111) and a Lynxeye linear position-sensitive. The powder patterns were recorded using a step size of 0.0197° across the angular range 10 ° ≤ 2θ ≤ 120 °. Structural analyses were performed by the Rietveld method using Jana2006 software^64^. Peak profile fitting was achieved musing Pseudo-Voigt functions.

### ^17^O MAS NMR

The spectra were acquired on a Bruker 9.4 T at the Larmor frequency of 54.25 MHz. To obtain quantitative results for La_2_Zr_2_O_7_ and Sm_2_Zr_2_O_7_, the spectra were acquired using a one pulse experiment with a very short length of 1 μs^65^. For Eu_2_Zr_2_O_7_ and Nd_2_Zr_2_O_7_, a Hahn echo was used with pulse durations of 3 μs (π/2) and 6 μs (π) with an echo delay of 16.3 μs (1 rotor period). In addition, two offsets were needed to acquire the full spectrum (to be sure that all the peaks were detected, we performed the experiments with several other offsets but here, we only show at the resonating frequency of the two relevant peaks). Because a Hahn echo experiment was used and the spectra acquired at two offsets, the data are only qualitative. All spectra were externally referenced to ^17^O enriched H_2_O set at 0 ppm. They were fitted with the DMfit software^66^ using the quad first model and the quadrupolar coupling constant, C_Q_, and the quadrupolar asymmetry parameter, η_Q_, were extracted from the 1D MAS NMR spectra.

### Raman spectroscopy

The experiments were performed at room temperature with a Horiba Jobin–Yvon T64000 spectrometer equipped with 1800 gr/mm grating. A 100× objective was used to focus the incident laser operating at 647 nm and collect the backscattered light. Extreme care was taken to avoid sample damage or laser induced heating. Measurements were performed at ~1 mW incident power. No significant change in the spectra was observed in this power range. It must be pointed out that as the samples are enriched in ^17^O, the isotopic shifts for all bands is about −1 to −2% which is in very good agreement with the calculated shift for one-phonon excitation and partial substitution (20 to 30% ^17^O enrichment) according to the harmonic model: $\frac{\bigtriangleup {\omega }_{i}}{{\omega }_{i}}=1-{(\frac{m{16}_{O}}{(1-x)m{16}_{O}+m{17}_{O}})}^{1/2}$where ${\omega }_{i}$ is the phonon frequency.

### Data availability statement

The datasets generated and/or analyzed during the current study are available from the corresponding author on reasonable request.

## Electronic supplementary material

Fingerprint of local disorder in long range ordered isometric pyrochlores
