# Supplementary material for: Fingerprint of local disorder in long range ordered isometric pyrochlores
Source: Sci Rep. 2017 Sep 25;7:12269. doi: 10.1038/s41598-017-12544-8 (PMC5613007; doi:10.1038/s41598-017-12544-8)
Supplement: Supplementary file 1 — Fingerprint of local disorder in long range ordered isometric pyrochlores [file 41598_2017_12544_MOESM1_ESM.doc]

**Supplementary Information**

Fingerprint of local disorder in long range ordered isometric pyrochlores

Laura Martel*, Mohamed Naji, Karin Popa, Jean-François Vigier and Joseph Somers

*European Commission, DG Joint Research Centre-JRC, Directorate G - Nuclear Safety and Security, Postfach 2340, D-76125 Karlsruhe, Germany*


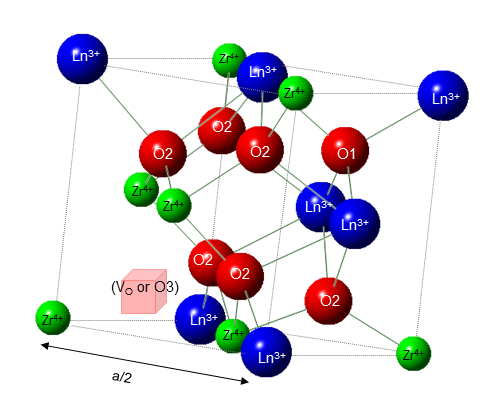


Supplementary Figure 1: The Ln_2_Zr_2_O_7_ ordered-pyrochlore (Ln=La, Nd, Sm and Eu) unit cell is represented. The structure possesses two different O crystallographic sites (O1, 8b) and (O2, 48f). An additional oxygen vacant site (V_O_, 8a) does exist and can be occupied by an extra (O3, 8a) site when substituted with another oxygen atom for charge balance.


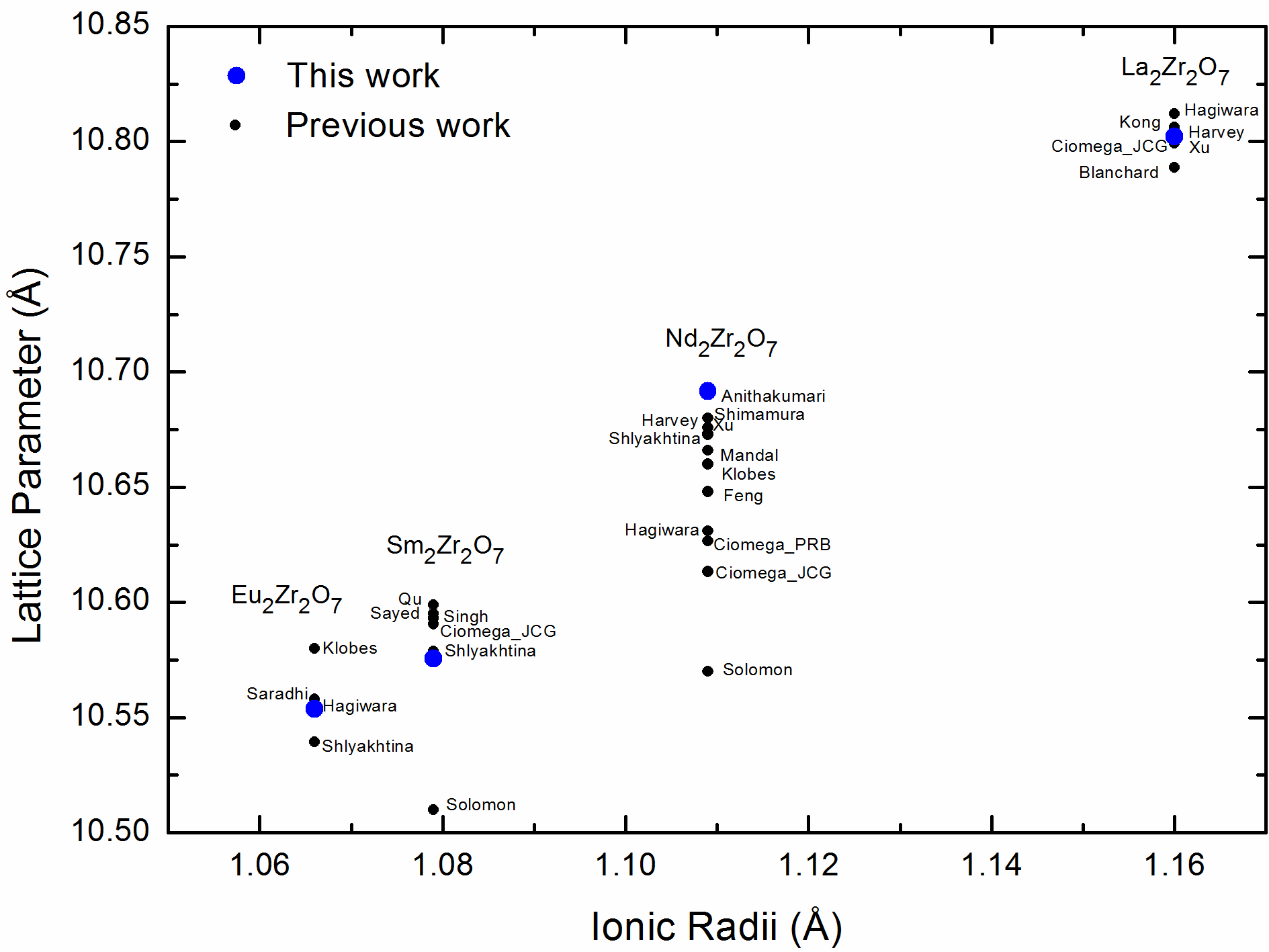


Supplementary Figure 2: Comparison of lattice parameters from the present work and previously published [[1]](#endnote-2),[[2]](#endnote-3),[[3]](#endnote-4),[[4]](#endnote-5),[[5]](#endnote-6),[[6]](#endnote-7),[[7]](#endnote-8),[[8]](#endnote-9),[[9]](#endnote-10),[[10]](#endnote-11),[[11]](#endnote-12),[[12]](#endnote-13) for the Ln_2_Zr_2_O_7_ plotted against their ionic radii[[13]](#endnote-14) (with r(Nd^3+^)=1.109 Å, r(Sm^3+^)=1.079 Å and r(Eu^3+^)=1.066 Å, r(Zr^4+^)=0.72 Å).


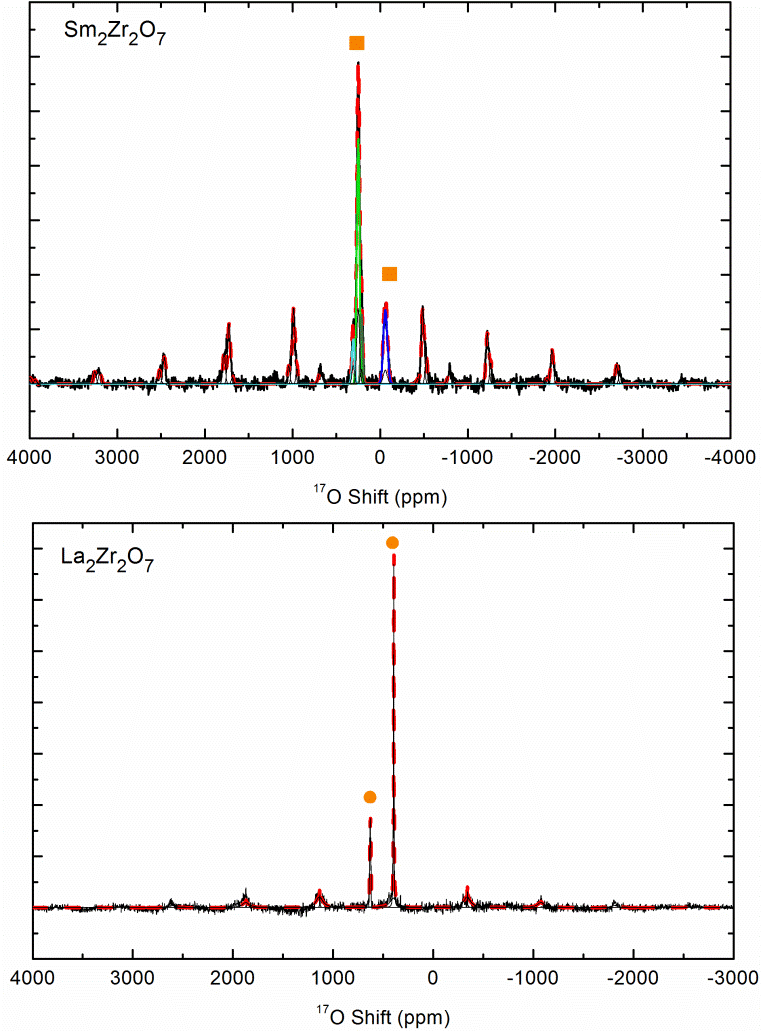


Supplementary Figure 3: Full ^17^O MAS NMR spectra and fits of La_2_Zr_2_O_7_ and Sm_2_Zr_2_O_7_ acquired at 40 kHz. The square and circle stand for the central transition peaks.


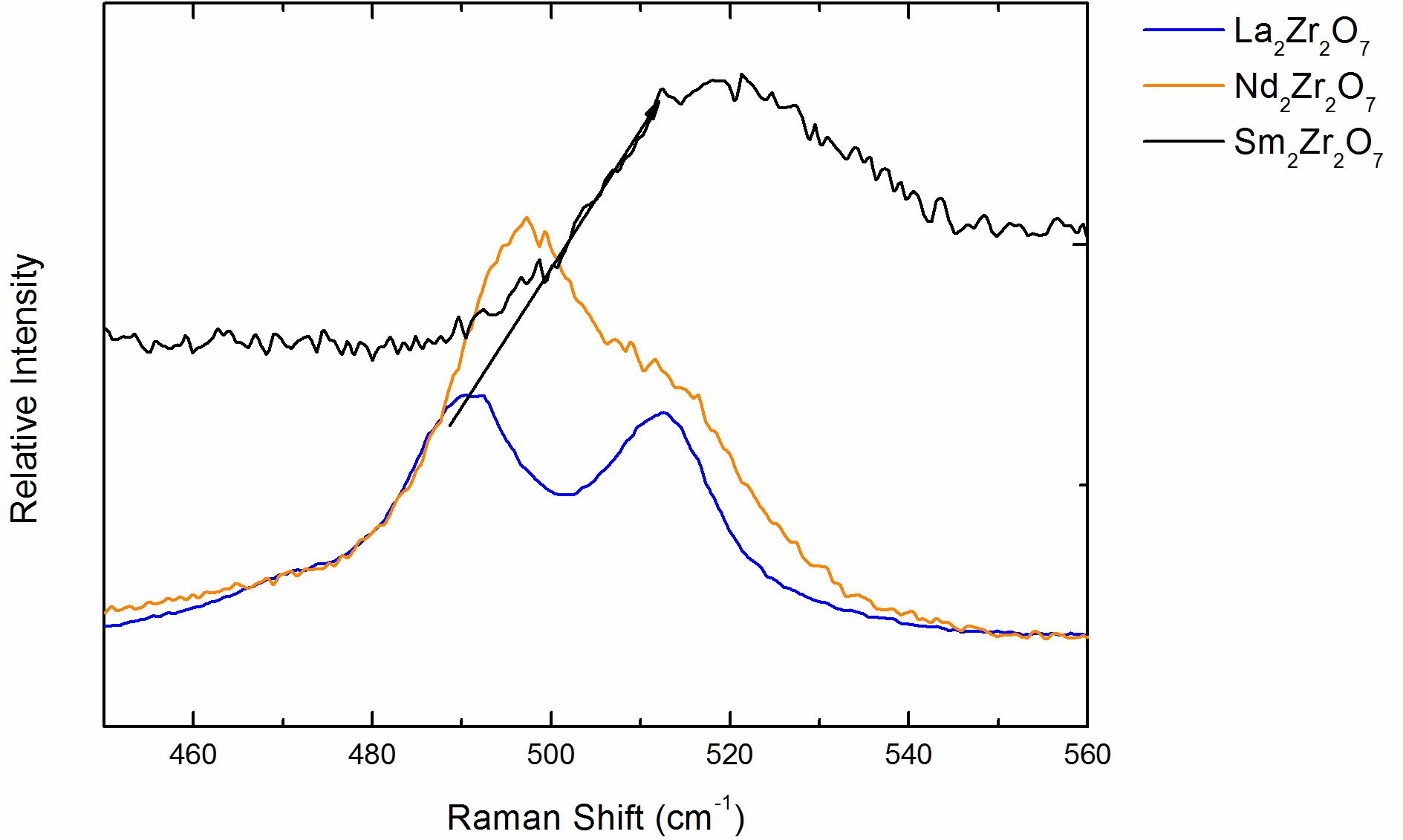


Supplementary Figure 4: Upshift of band E*in La_2_Zr_2_O_7_ and Nd_2_Zr_2_O_7_ while the band F* is almost constant and falls into the same position as band G* in Sm_2_Zr_2_O_7_.

1. Supplementary note 1: Understanding the ^17^O NMR shifts

To empirically confirm the NMR shift attribution, and as previously suggested by Kim and Grey[[14]](#endnote-15), we use the ^17^O isotropic chemical shifts previously published for La_2_O_3_[[15]](#endnote-16). In fact, as O1 is only surrounded by four La (OLa_4_), its ^17^O isotropic chemical shift should be similar to the one of 584 ppm determined for the OLa_4_ in La_2_O_3_. Fortunately, it is exactly what we observed here confirming the attribution of peak A to these species. We also noticed that for all the Ln_2_Zr_2_O_7_ with lanthanide possessing unpaired electrons (Nd, Sm and Eu), very similar ^17^O NMR shifts are found between their OLn_4_ (O1, 8b) sites and the one of the Ln_2_O_3_ series (Supplementary Figure 5)[[16]](#endnote-17) even if their crystalline structure are different. In addition, for the OLn_4_ peak in Nd_2_Zr_2_O_7_ (Supplementary Figure 6) and Eu_2_Zr_2_O_7_ (Supplementary Figure 7), the ^17^O NMR shift also varies strongly with the spinning rate (Supplementary Table 1) due to a slight heating of the sample[[17]](#endnote-18),[[18]](#endnote-19) confirming the paramagnetic effect.


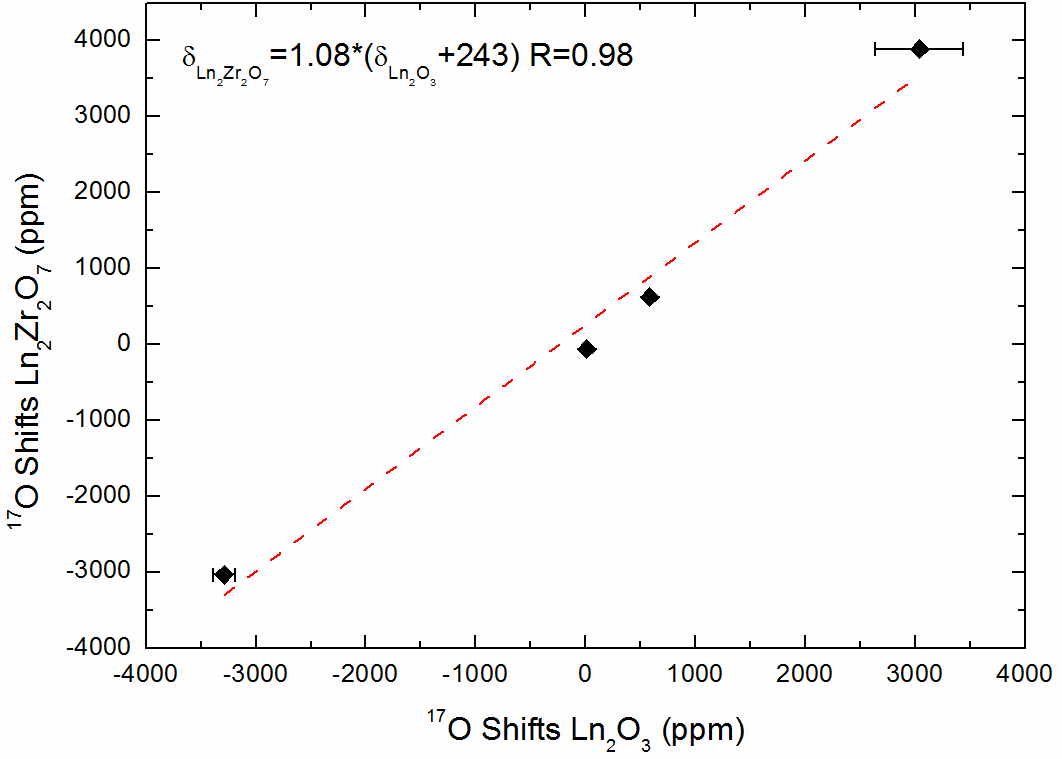


Supplementary Figure 5: Plot between the ^17^O shifts of the lanthanide pyrochlores (Ln_2_Zr_2_O_7_) and the lanthanide sesquioxidesError: Reference source not found (Ln_2_O_3_).


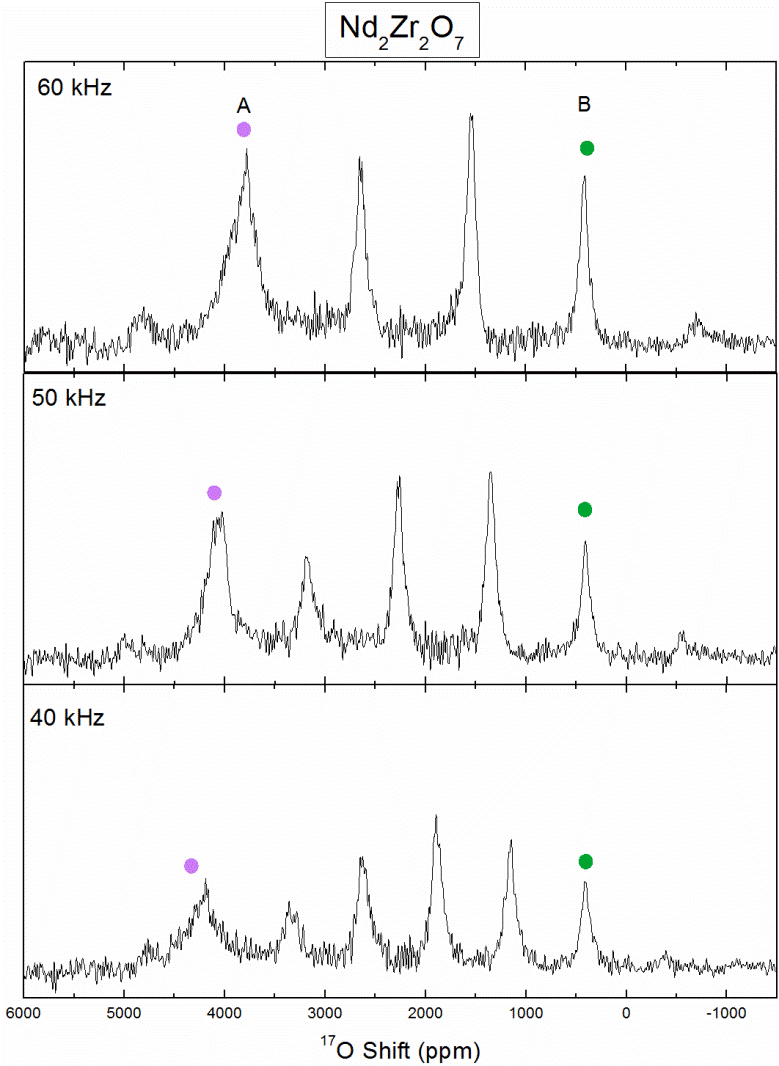


Supplementary Figure 6: ^17^O MAS NMR spectra of Nd_2_Zr_2_O_7_ acquired at three spinning rates (offset 3000 ppm).


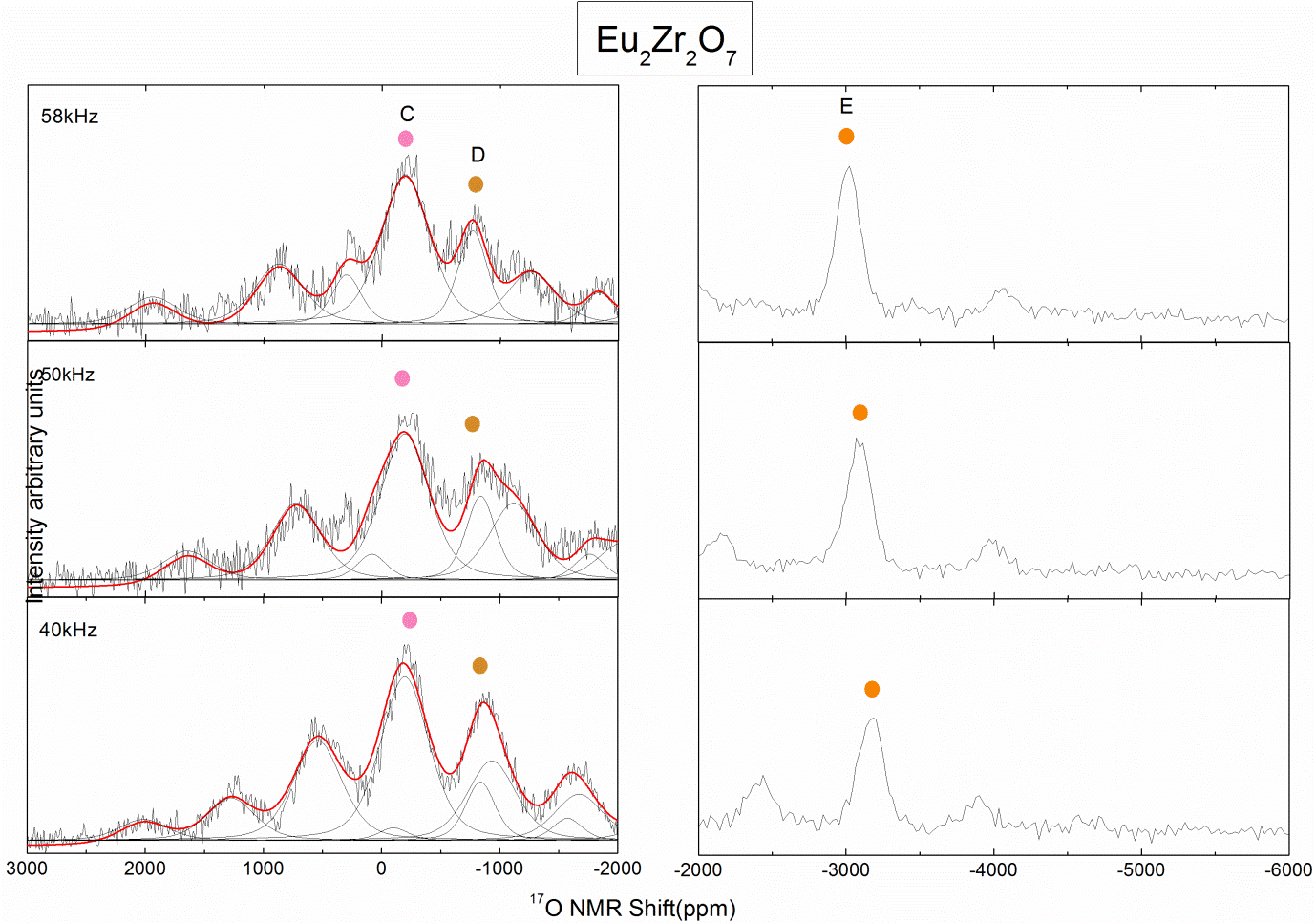


Supplementary Figure 7: ^17^O MAS NMR spectra of Eu_2_Zr_2_O_7_ acquired at three spinning rates and two different offsets (Left -230 ppm and right -3000 ppm).

Supplementary Table 1: Variation of the ^17^O Shifts with spinning rate.

|  | ^17^O Shifts (ppm) | | |
| --- | --- | --- | --- |
|  | Peak A | Peak E | Peak I |
| Spinning rate (kHz) |  |  |  |
| 40 | -60.3 | 4291 | -3188 |
| 50 |  | 4127 | -3093 |
| 58 |  |  | -3035 |
| 60 |  | 3890 |  |

1. Supplementary note 2: Order and disorder through the Ln_2_Zr_2_O_7_ series


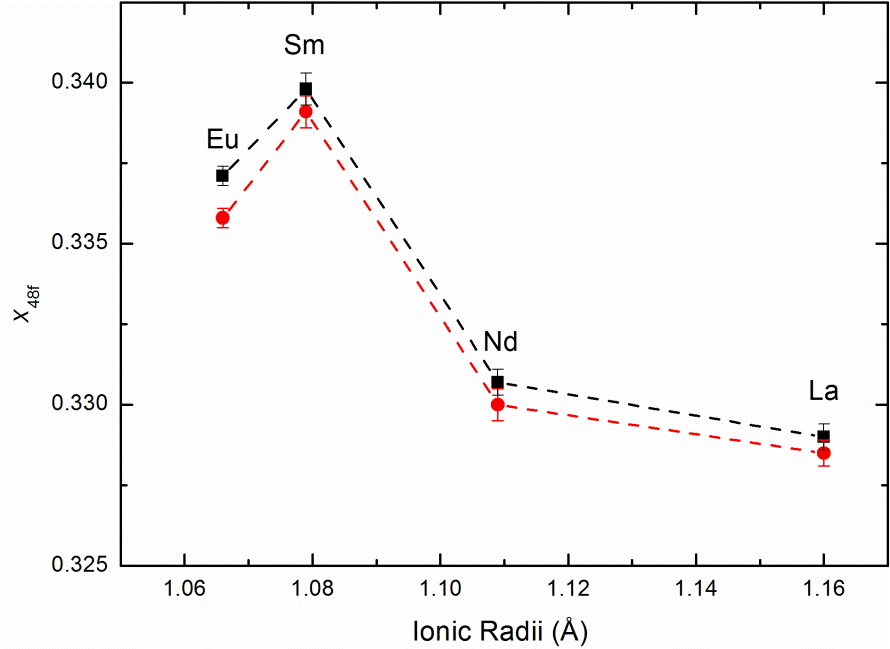


Supplementary Figure 8: Variation of the *x*_48f_ parameter (square when considering an ordered pyrochlore with 2O and circle when considering long range disorder with 3O) as a function of the ionic radii. The dashed lines are a guide for the eye.

As XRD is the most commonly used technique to characterize the long range disorder through the lanthanide pyrochlore series, Rietvield refinements were performed for all the patterns. Two models were considered: *i)* the ordered crystalline structure with two oxygen sites (O1, 8b) and (O2, 48f) (Supplementary Table 2 and Supplementary Table 3); and *ii)* the disordered crystalline structure with three oxygen sites (O1, 8b), (O2, 48f) and (O3, 8a) (Supplementary Table 4 and Supplementary Table 5) (A third model using cation swapping was also used, but, the best model for the reﬁnements is the one in which there is no mixed occupancy of the cationic sites and no cationic/anionic deﬁciencies could be detected (not shown). Moreover, ﬁtting these data using different models discussed in literature for the occupancies of the cations sites showed that there is no deﬁciency in Ln^3+^ or Zr^4+^ content, and no internal site disorder was detected in our materials (at the long range order). The first model is nevertheless favoured as while neutron diffraction is very appropriate to detect the O atoms, due to the presence of the two heavy cations, XRD is not as well suited. [[19]](#endnote-20),[[20]](#endnote-21) A clear example is the case of Eu_2_Zr_2_O_7_ for which, by introducing disorder in the Rietvield refinement, Hagiwara et al.Error: Reference source not found determined an occupancy for O3 of about 0.47 while, with the same lattice parameter, we obtained here a higher occupancy of 0.75. Thus, with the model free of disorder, we determined the *x*_48f_-parameter atomic coordinate as it is often considered as a good probe of disorder.Error: Reference source not found, [[21]](#endnote-22) We consequently plotted it as a function of the lanthanide cation in Supplementary Figure 8. Unfortunately, its variation is not straightforward and, at first sight, no specific trend or change of slope can be extracted.

Increasing disorder through the series can be investigated using Raman spectroscopy as shown by Scheetz and White who described a spectral line broadening accompanied with an intensity decrease though the lanthanide zirconate pyrochlore series (Eu_2_Zr_2_O_7_ was not studied by the authors)[[22]](#endnote-23). In our study, we took the opportunity to confirm this observation through the increase of the line broadening -expressed by its full width at half maxima (FWHM)- and the decrease in intensities as portrayed in Figure 4. As going from La to Eu, there is a broadening of the B* and D* bands as well as a decrease in their intensities. The broadening of the Raman bands may be caused by several factors such as an inherent disorder,[[23]](#endnote-24) a decrease in particle size[[24]](#endnote-25), or non-harmonic effects.[[25]](#endnote-26),[[26]](#endnote-27) Regarding the particle size, the XRD patterns show sharp lines (Figure 1), which then excludes a broadening due to a smaller particle size. Anharmonic effects can be also excluded since literature results from Raman spectroscopy measurements showed that anharmonicity is slightly contributing to high energy modes at room temperature[[27]](#endnote-28). Therefore, the band broadening for Sm_2_Zr_2_O_7_ and Eu_2_Zr_2_O_7_ as compared to Nd_2_Zr_2_O_7_ and La_2_Zr_2_O_7_ can only be attributed to an inherent disorder in these systems. Disorder disrupts the translational symmetry in the lattice and consequently relaxes the Q≈0 selection rule. Hence, phonons from all parts of the Brillouin zone start contributing to the optical spectra, thereby giving rise to broadened, continuously spread, and weak-intensity bands[[28]](#endnote-29). Note that the decrease in Raman intensity is usually correlated (if the numbers of oscillators is constant) with the increase of the band-width (and vice-versa), as both parameters are linearly related through a Lorentzien function.

Supplementary Table 2: Refined structural parameters for the three lanthanide pyrochlores considering the non-disordered pyrochlore.

|  | atom | Wyck. | occupancy | x | y | z | U_iso_^1^ |
| --- | --- | --- | --- | --- | --- | --- | --- |
| La_2_Zr_2_O_7_ | La1 | 16d | 1 | 0.5 | 0.5 | 0.5 | 0.0090(3) |
|  | Zr1 | 16c | 1 | 0 | 0 | 0 | 0.0052(3) |
|  | O1 | 8b | 1 | 0.625 | 0.625 | 0.625 | 0.0035(12) |
|  | O2 | 48f | 1 | 0.3290(4) | 0.125 | 0.125 | 0.0035(12) |
| Nd_2_Zr_2_O_7_ | Nd1 | 16d | 1 | 0.5 | 0.5 | 0.5 | 0.0042(4) |
|  | Zr1 | 16c | 1 | 0 | 0 | 0 | 0.0033(4) |
|  | O1 | 8b | 1 | 0.625 | 0.625 | 0.625 | 0.0037(16) |
|  | O2 | 48f | 1 | 0.3307(4) | 0.125 | 0.125 | 0.0037(16) |
| Sm_2_Zr_2_O_7_ | Sm1 | 16d | 1 | 0.5 | 0.5 | 0.5 | 0.0027(5) |
|  | Zr1 | 16c | 1 | 0 | 0 | 0 | 0.0062(5) |
|  | O1 | 8b | 1 | 0.625 | 0.625 | 0.625 | 0.0100^2^ |
|  | O2 | 48f | 1 | 0.3398(5) | 0.125 | 0.125 | 0.0100 |
| Eu_2_Zr_2_O_7_ | Eu1 | 16d | 1 | 0.5 | 0.5 | 0.5 | 0.0194(5) |
|  | Zr1 | 16c | 1 | 0 | 0 | 0 | 0.0065(4) |
|  | O1 | 8b | 1 | 0.625 | 0.625 | 0.625 | 0.0100^2^ |
|  | O2 | 48f | 1 | 0.3371(3) | 0.125 | 0.125 | 0.0100 |
| ^1^U_iso_ of oxygen atoms are considered equal | | | | | | | |
| ^2^Not refined since refinement were giving unrealistically low value | | | | | | | |

Supplementary Table 3: Refined crystallographic parameters for the three lanthanide pyrochlores obtained using a Rietveld refinement considering the non-disordered pyrochlore.

| Formula | La_2_Zr_2_O_7_ | Nd_2_Zr_2_O_7_ | Sm_2_Zr_2_O_7_ | Eu_2_Zr_2_O_7_ |
| --- | --- | --- | --- | --- |
| Z | 8 | 8 | 8 | 8 |
| Lattice parameter (Å) | 10.8019(1) | 10.6911(1) | 10.5753(1) | 10.5533(1) |
| Rp | 3.40 | 2.48 | 2.06 | 1.69 |
| Rwp | 5.76 | 3.92 | 3.29 | 2.45 |
| Goodness of Fit | 3.05 | 2.01 | 1.96 | 1.47 |

Supplementary Table 4: Refined structural parameters for the three lanthanide pyrochlores considering a partial filling of the O3 site. The charge balance is maintained by transferring the anion from the 48f site to the vacant 8a site.

|  | atom | Wyck. | occupancy | x | y | z | U_iso_^1^ |
| --- | --- | --- | --- | --- | --- | --- | --- |
| La_2_Zr_2_O_7_ | La1 | 16d | 1 | 0.5 | 0.5 | 0.5 | 0.0090(3) |
|  | Zr1 | 16c | 1 | 0 | 0 | 0 | 0.0056(4) |
|  | O1 | 8b | 1 | 0.625 | 0.625 | 0.625 | 0.0027(12) |
|  | O2 | 48f | 0.982(3) | 0.3285(4) | 0.125 | 0.125 | 0.0027(12) |
|  | O3 | 8a | 0.10(2) | 0.125 | 0.125 | 0.125 | 0.0027(12) |
| Nd_2_Zr_2_O_7_ | Nd1 | 16d | 1 | 0.5 | 0.5 | 0.5 | 0.0044(4) |
|  | Zr1 | 16c | 1 | 0 | 0 | 0 | 0.0029(4) |
|  | O1 | 8b | 1 | 0.625 | 0.625 | 0.625 | 0.0025(16) |
|  | O2 | 48f | 0.976(4) | 0.3300(5) | 0.125 | 0.125 | 0.0025(16) |
|  | O3 | 8a | 0.13(3) | 0.125 | 0.125 | 0.125 | 0.0025(16) |
| Sm_2_Zr_2_O_7_ | Sm1 | 16d | 1 | 0.5 | 0.5 | 0.5 | 0.0031(5) |
|  | Zr1 | 16c | 1 | 0 | 0 | 0 | 0.0084(5) |
|  | O1 | 8b | 1 | 0.625 | 0.625 | 0.625 | 0.0100^2^ |
|  | O2 | 48f | 0.925(5) | 0.3391(5) | 0.125 | 0.125 | 0.0100 |
|  | O3 | 8a | 0.45(3) | 0.125 | 0.125 | 0.125 | 0.0100 |
| Eu_2_Zr_2_O_7_ | Eu1 | 16d | 1 | 0.5 | 0.5 | 0.5 | 0.0179(5) |
|  | Zr1 | 16c | 1 | 0 | 0 | 0 | 0.0109(4) |
|  | O1 | 8b | 1 | 0.625 | 0.625 | 0.625 | 0.0100^2^ |
|  | O2 | 48f | 0.876(3) | 0.3358(3) | 0.125 | 0.125 | 0.0100 |
|  | O3 | 8a | 0.75(2) | 0.125 | 0.125 | 0.125 | 0.0100 |
| ^1^U_iso_ of oxygen atoms are considered equal | | | | | | | |
| ^2^Not refined since refinement were giving unrealistically low value | | | | | | | |

Supplementary Table 5: Refined crystallographic parameters for the three lanthanide pyrochlores obtained using a Rietveld refinement considering a partial filling of the O3 site.

| Formula | La_2_Zr_2_O_7_ | Nd_2_Zr_2_O_7_ | Sm_2_Zr_2_O_7_ | Eu_2_Zr_2_O_7_ |
| --- | --- | --- | --- | --- |
| Lattice parameter (Å) | 10.8019(1) | 10.6911(1) | 10.5753(1) | 10.5534(1) |
| Rp | 3.40 | 2.48 | 2.05 | 1.66 |
| Rwp | 5.75 | 3.92 | 3.26 | 2.31 |
| GOF | 3.05 | 2.00 | 1.94 | 1.38 |

Supplementary note 3: Nutation experiment and O local distortion


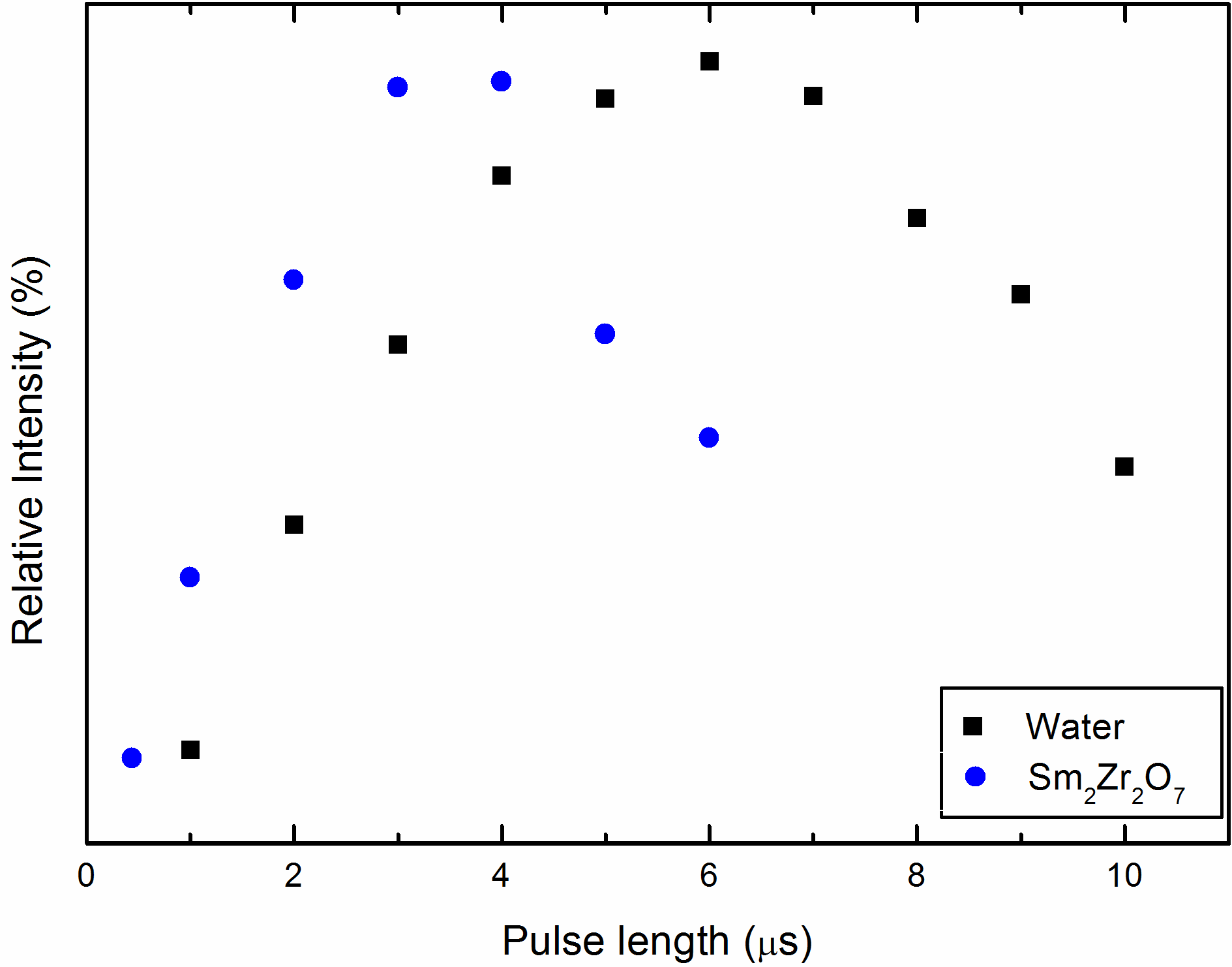


Supplementary Figure 9: ^17^O nutation experiment performed on water and the peak detected at -60.3 ppm on the Sm_2_Zr_2_O_7_ spectrum.

As ^17^O is a quadrupolar nucleus (i.e., spin 5/2), presence of local bond angle distortions around the oxygen atoms will create appearance of quadrupole interaction[[29]](#endnote-30) (i.e., a quadrupolar coupling constant (C_Q_) not null) which is here characterized by a pattern of spinning sidebands. To verify if such distortion exists, nutation experiments that consist of the observation of the center band magnetization variation with increasing 90° pulse lengths (τ_90°_) can be done especially when dealing with small C_Q_.[[30]](#endnote-31) By comparing the nutation curve of a given compound with that of a reference sample, known with C_Q_ = 0 (here enriched-^17^O water), one can determine if C_Q_ ≠ 0 (i.e., different variation of the nutation curves). It is exactly what we observe here in Supplementary Figure 9 proving the presence of local distortion around the (O1, 8b) atom. For Eu_2_Zr_2_O_7_, due to an important spectral broadening, the acquisition of a single pulse experiment leads to a non-exploitable spectrum and the same type of nutation experiment was unfortunately not possible. Nevertheless, we do believe that the additional spinning sidebands for peak K are caused by the same effect.

**References**

1. Hagiwara, T., Yamamura, H., Nomura, K., Igawa, M. Relationship between crystal structure and oxide-ion conduction in Ln_2_Zr_2_O_7_ (Ln = Eu, Nd and La) system deduced by neutron and X-ray diffraction, *J. Ceram. Soc. Jpn*, **121**, 205-210 (2013). [↑](#endnote-ref-2)
2. Shlyakhtina, A. V., Belov, D. A., Knotko, A. V., Kolbanev, I. V., Streletskii, A. N., Karyagina, O. K., Shcherbakova, L. G., Oxygen interstitial and vacancy conduction in symmetric Ln_2 ± x_Zr_2 ± x_O_7 ± x/2_ (Ln = Nd, Sm) solid solutions, *Inorg. Mater.*, **50**, 1035–1049 (2014). [↑](#endnote-ref-3)
3. Finkeldei, S. C., PhD Thesis, Pyrochlore as nuclear waste form: actinide uptake and chemical stability, Jülich Forchungszentrum, Nov. 2014. [↑](#endnote-ref-4)
4. Anithakumari, P., Grover, V., Nandi, C., Bhattacharyya, K., Tyagia, A. K., Utilizing non-stoichiometry in Nd_2_Zr_2_O_7_ pyrochlore: exploring superior ionic conductors, *RSC Adv.*, **6**, 97566-97579 (2016). [↑](#endnote-ref-5)
5. Singh, S., Saha, S., Dhar, S. K., Suryanarayanan, R., Sood, A. K., Revcolevschi, A., Manifestation of geometric frustration on magnetic and thermodynamic properties of the pyrochlores Sm_2_X_2_O_7_ (X = Ti, Zr), *Phys. Rev. B*, **77**, 054408 (2008). [↑](#endnote-ref-6)
6. Van Dijk, M. P., Ter Maat, J. H. H., Roelofs, G., Bosch, H., Van de Velde, G.M.H., Gellings, P. J., Burggraaf, A. J., Electrical and catalytic properties of some oxides with the fluorite or pyrochlore structure, *Mat. Res. Bull.*, **19**, 1149-1156 (1984). [↑](#endnote-ref-7)
7. Ciomaga Hatnean, M., Lees, M. R., Petrenko, O. A., Keeble, D. S., Balakrishnan, G., Gutmann, M. J., Klekovkina, V. V., Malkin, B. Z., Structural and magnetic investigations of single-crystalline neodymium zirconate pyrochlore Nd_2_Zr_2_O_7_, *Phys. Rev. B*, **91**, 174416 (2015). [↑](#endnote-ref-8)
8. Kong, L., Karatchevtseva, I., Gregg, D. J., Blackford, M. G., Holmes, R., Triani, G., A novel chemical route to prepare La_2_Zr_2_O_7_ pyrochlore, *J. Am. Ceram. Soc.*, **96**, 935–941 (2013). [↑](#endnote-ref-9)
9. Ciomaga Hatnean, M., Lees, M. R., Balakrishnan, G., Growth of single-crystals of rare-earth zirconate pyrochlores, Ln_2_Zr_2_O_7_ (with Ln = La, Nd, Sm, and Gd) by the floating zone technique *J. Crys. Growth*, **418**, 1–6 (2015). [↑](#endnote-ref-10)
10. Klobes, B., Finkeldei, S., Röhrig, W., Bosbach, D., Hermann, R. P., Hyperfine interactions in and lattice parameters of pyrochlore and defect fluorite (Eu_1-x_Nd_x_)_2_Zr_2_O_7_, *J. Phys. Chem. Solids*, **79**, 43–48 (2015). [↑](#endnote-ref-11)
11. Qu, Z., Wan, C., Pan, W., Thermal Expansion and Defect Chemistry of MgO-Doped Sm_2_Zr_2_O_7_, *Chem. Mater.*, **19**, 4913-4918 (2007). [↑](#endnote-ref-12)
12. S. Solomon, A. George, J. K. Thomas, and A. John, Preparation, characterization, and ionic transport properties of nanoscale Ln_2_Zr_2_O_7_ (Ln = Ce, Pr, Nd, Sm, Gd, Dy, Er, and Yb) Energy Materials, *J. Elec. Mater.*, **44**, 28-37 (2015). [↑](#endnote-ref-13)
13. R.D. Shannon, Revised effective ionic radii and systematic studies of interatomic distances in halides and chaleogenides, Acta Cryst., A32, 751–767 (1976). [↑](#endnote-ref-14)
14. Kim, N., Grey, C. P., ^17^O MAS NMR study of the oxygen local environments in the anionic conductors Y_2_(B_1-x_B'_x_)_2_O_7_(B; B' = Sn; Ti, Zr), *J. Solid State Chem.*, **175**, 110-115 (2003). [↑](#endnote-ref-15)
15. Bastow, T.J., Stuart, S.N., ^17^O NMR in simple oxides, *Chem. Phys.*, **143**, 459-467 (1990). [↑](#endnote-ref-16)
16. Yang, S., Shore, J., Oldfield, E., Oxygen-17 Nuclear magnetic resonance spectroscopic study of the lanthanide oxides, *J. Magn. Reson.*, **99**,408-412 (1992). [↑](#endnote-ref-17)
17. Grey, C.P., Cheetham, A.K., Dobson, C.M., Temperature-dependent solid-state 119Sn- MAS NMR of Nd_2_Sn_2_O_7_, Sm_2_Sn_2_O_7_, and Y_1.8_Sm_0.2_Sn_2_O_7_ three sensitive chemical-shift thermometers, *J. Magn. Res. A*, **101**, 299-306 (1993). [↑](#endnote-ref-18)
18. Martel, L., Magnani, N., Boshoven, J., Vigier, J.-F., Selfslag, C., Farnan, I., Griveau, J.-C., Somers, J., Fanghänel T., High-resolution solid-state oxygen-17 NMR of actinide-bearing compounds: An insight into the 5f chemistry, *Inorg. Chem.*, **5**3, 6928−6933 (2014). [↑](#endnote-ref-19)
19. Blanchard, P. E. R., Clements, R., Kennedy, B. J., Ling, C. D., Reynolds, E., Avdeev, M., Stampfl, A. P. J., Zhang, Z., Jang, L.-Y., Does Local Disorder Occur in the Pyrochlore Zirconates?, *Inorg. Chem.*, **51**, 13237−13244 (2012). [↑](#endnote-ref-20)
20. R. Clements, J. R.Hester, B. J. Kennedy, C. D. Ling, A. P. J. Stampfl, The fluorite–pyrochlore transformation of Ho_2-y_Nd_y_Zr_2_O_7_, *J. Solid State Chem.*, **184**, 2108–2113 (2011). [↑](#endnote-ref-21)
21. Mandal, B. P., Krishna, P. S. R., Tyagi, A. K., Order–disorder transition in the Nd_2-y_Y_y_Zr_2_O_7_ system: Probed by X-ray diffraction and Raman spectroscopy, *J. Solid State Chem.*, **183**, 41–45 (2010). [↑](#endnote-ref-22)
22. Scheetz, B. E., White, W. B., Characterization of anion disorder in zirconate A_2_B_2_O_7_ compounds by Raman spectroscopy, *J. Am. Ceram. Soc.*, **62**, 468-470 (1979). [↑](#endnote-ref-23)
23. White; W. B., pp. 87- 1 I0 in The Infrared Spectra of Minerals. Edited b) V. C. Farmer. Mineralogical Society, London. 1974. [↑](#endnote-ref-24)
24. Michel, D., Perez y Jorba, M., Collongues, R., study by raman spectroscopy of order-disorder phenomena occurring in some binary oxides with fluorite-related structures, *J. Raman Spectrosc.*, **5** 163-180 (1976). [↑](#endnote-ref-25)
25. Paul, B., Singh, K., Jarón, T., Roy, A., Chowdhury A., Structural properties and the fluorite-pyrochlore phase transition in La_2_Zr_2_O_7_: The role of oxygen to induce local disordered states, *J. Alloys. Compd*, **686**, 130-136 (2016). [↑](#endnote-ref-26)
26. Zhang, F. X., Manoun, B., Saxena, S. K., Zha, C. S., Structure change of pyrochlore Sm_2_Ti_2_O_7_ at high pressures, *Appl. Phys. Lett.*, **86**, 181906 (2005). [↑](#endnote-ref-27)
27. Mandal, B.P, Banerji, A., Sathe, V., Deb, S. K., Tyagi, A. K., Order–disorder transition in Nd_2-y_Gd_y_Zr_2_O_7_ pyrochlore solid solution: An X-ray diffraction and Raman spectroscopic study, *J. Solid State Chem.*, **180**, 2643-2648 (2007). [↑](#endnote-ref-28)
28. Cardona, M., Merlin, R., Light scattering in solids IX. Springer Berlin Heidelberg, 2006, 1-14. [↑](#endnote-ref-29)
29. Kentgens, A. P. M. A practical guide to solid-state NMR of halfinteger quadrupolar nuclei with some applications to disordered systems, *Geoderma*, **80**, 271−30 (1997). [↑](#endnote-ref-30)
30. Freude, D.; Haase, J. Quadrupole effects in solid-state nuclear magnetic resonance. NMR Basic Principles Prog. 1993, 29, 1−90. [↑](#endnote-ref-31)
